# Towards Accurate Node-Based Detection of P2P Botnets

**DOI:** 10.1155/2014/425491

**Published:** 2014-06-24

**Authors:** Chunyong Yin

**Affiliations:** ^1^School of Computer & Software, Nanjing University of Information Science & Technology, Nanjing 210044, China; ^2^Jiangsu Engineering Center of Networking Monitoring, Nanjing University of Information Science & Technology, Nanjing 210044, China

## Abstract

Botnets are a serious security threat to the current Internet infrastructure. In this paper, we propose a novel direction for P2P botnet detection called node-based detection. This approach focuses on the network characteristics of individual nodes. Based on our model, we examine node's flows and extract the useful features over a given time period. We have tested our approach on real-life data sets and achieved detection rates of 99-100% and low false positives rates of 0–2%. Comparison with other similar approaches on the same data sets shows that our approach outperforms the existing approaches.

## 1. Introduction

### 1.1. Background and Motivation

Botnets are groups of computers which are libked to each other through similar network processes which perform coordinated tasks like information crawling, Internet Relay Chatting (IRC), and information sharing. While botnets can be used for benign purposes, over the past decade, there has been a drastic increase in the number of malicious botnets [1] which have become a serious concern to the security of Internet applications and networking infrastructure. To build a malicious botnet, the attacker, known as botmaster, uses one or more central servers to compromise and acquire control of vulnerable computers using malware. These compromised computers, known as “bots,” link to the central server, which acts as the command and control center (C&C) using protocols like IRC or HTTP. The botmaster uses these channels to deliver additional malware and instructions to the bots for launching different kinds of attacks. Malicious botnets are capable of a wide range of attacks including e-mail spam, keystroke logging, packet sniffing, DoS attacks, and identifying new targets for enlisting in the botnet, among others [2–6]. In this paper, unless explicitly stated, we use the term “botnet” to refer to a malicious botnet.

**Figure 1:**
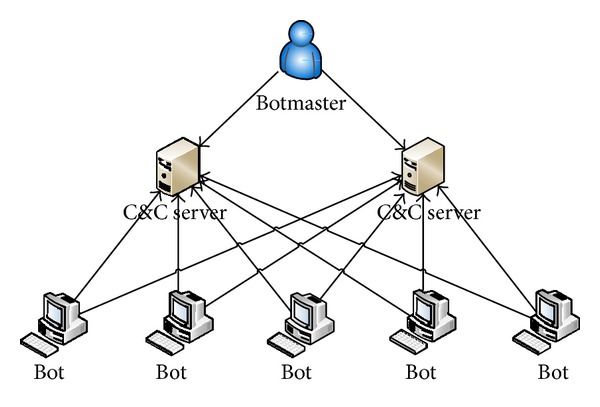
C&C botnet.

**Figure 2:**
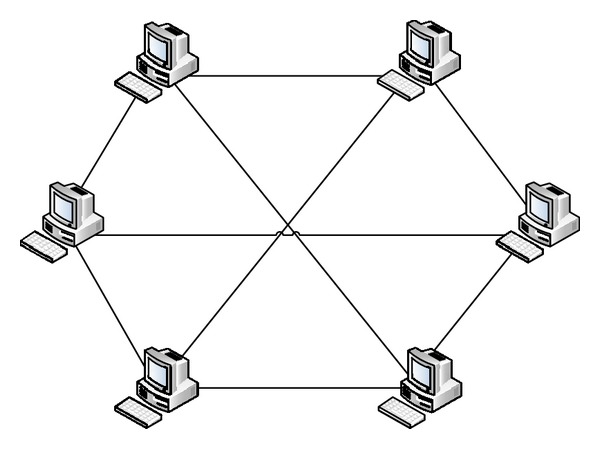
P2P network.

**Figure 3:**
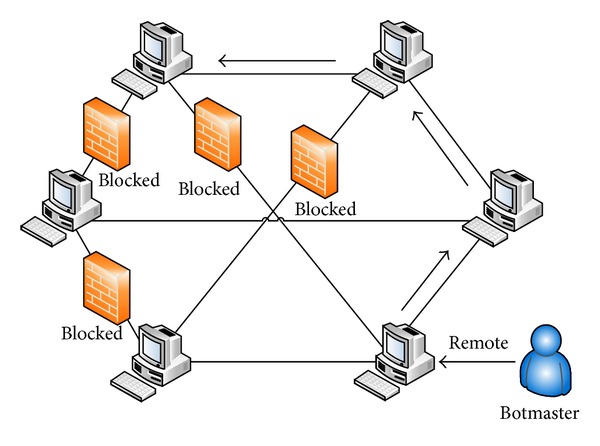
P2P botnet bypassing firewalls.

Figure 1 illustrates a botnet of five bots connected to two C&C servers controlled by a botmaster. This botnet is operating in a centralized mode; that is, one or two servers control the bots in the network. Such centralized botnets can be shutdown or blocked if the C&C servers are identified, thereby rendering the botnet ineffective. To increase resiliency to detection, recent botnets are built using peer-to-peer networking principles where any node can act as a client as well as a server. Accordingly, in a P2P botnet any node can act as a bot as well as the C&C server. In Figure 2, we show an example P2P network and in Figure 3 we show a corresponding P2P botnet. The botmaster can connect to any P2P bot in the network and operate it as the C&C server. Compared to the server-client botnet, the P2P botnet has the ability to realize highly scalable and extensible network structure which is resilient to firewall sanctions and node/path failures.

In this paper, we focus on the problem of detecting P2P bots in a distributed network. Mitigating the threat of a P2P botnet is a challenging task as the botnet has no central C&C server which can be blocked and the botmaster uses the overlay structure of the P2P network to stay connected to the bots. As shown in Figure 3, even if some bots are blocked by firewalls, the botmaster can continue communication with these bots along alternate routing paths as long as the blocked nodes are connected to at least one other P2P bot. This implies that it is essential to detect the bots in a systematic and comprehensive manner. Furthermore, the malware programs used in P2P bots are, typically, self-propagating—which help in discovering new peers and mutating as well—to avoid signature-based detection. Due to their resilience, P2P botnets represent a major threat to the Internet applications and infrastructure. Therefore, to safeguard the Internet from strategic coordinated attacks, there is an urgent need to devise solutions to detect P2P bots and render the P2P botnets ineffective.

### 1.2. Limitation of Prior Art

The existing solutions for P2P botnet detection can be broadly classified into signature [7–13] and flow-based detection [14, 15, 15–18]. Signature-based P2P bot detection is based on inspecting each packet in the network traffic, entering or leaving the Internet gateway of the network, for the presence of special features such as port numbers, byte sequences in the payload, and blacklisted IP address. These special features, known as signatures, are extracted from known botnet infections in the past and stored in a signature database. While signature-based detection has good detection rate and is easily deployed, it has two major limitations. First, it is deterministic and relies only on detecting known botnet infections and cannot detect unknown bots. Even known bots can evade signature detection by changing ports of communication or use packet payload encryption to hide the bot specific features. Second, inspection of each packet results in performance degradation especially when the traffic consists of a large amount of benign data.

Flow analysis based bot detection examines network flows between two nodes where a flow is defined as a set of packets which have the same source address, source port, destination address, and destination port. The intuition in these approaches is that the flow features, such as the count of the packets in the flow, the order of the packet arrivals, and the interval between packets, can model the botnet communication patterns more accurately than direct packet inspection. The extracted features are used to construct a classifier that can differentiate normal flows and malicious bot flows. Since classifiers use statistical profiling, flow-based analysis is capable of detecting unknown bots which exhibit behavioral similarities to known bots. However, flow-based techniques suffer from two key limitations. First, there are several flows between any two network nodes which need to be analyzed and, usually, most of these flows belong to normal network processes. Second, the flow features need to be extracted at runtime which implies that flow-based analysis requires considerable computational overhead at runtime. At any given instant, there are a significant number of flows in the network which exaggerate the impact of these limitations further.

### 1.3. Proposed Approach

In this paper, we describe a novel P2P bot detection approach, called node-based bot detection, in which we analyze the network profile of nodes to detect bot characteristics. A sample network profile of a node may comprise the different protocols used by the node, the number of flows in a particular time period, packet statistics, and so on. Our approach is based on the intuition that P2P bots exhibit a distinct network profile due to the various P2P network maintenance related tasks they are required to perform. A P2P bot will be more active in communicating with other P2P bots and exchanging various instructions related to control and command. Also, unlike normal P2P nodes, the P2P bots exhibit nearly uniform network activity based on the instructions of the current C&C server. Based on these observations, our approach consists of identifying and quantifying the network profile features that are typical of a P2P bot. To extract these features, we monitor the network flows at each node and generate the node's network profile. The final network profile of a node is a combination of the features typical of P2P bots and the features observed from the network flows at the node. Finally, we use machine learning based classification techniques to detect whether the network profile of a node corresponds to the network profile of a P2P bot.

### 1.4. Technical Challenges and Solutions

There are several technical challenges in our approach. First, the process of constant flow monitoring at a node results in a major computational and storage overhead. We address this issue by using a sampling approach, wherein we periodically sample a network flow at different time intervals. Although sampling may not detect the same number of bots as those detected by constant flow monitoring in the same time interval, due to the cyclic nature of P2P botnets, the sampling approach eventually detects all the bots in the P2P botnet. Second, quantifying the network profile of P2P bots is nontrivial as different botnets exhibit different semantics and use variable protocols. To address this concern, we abstract and model the general network features of a P2P botnet using the profiles of few existing P2P botnets. We focus on the communication patterns of P2P botnets and do not consider the individual protocol and payload features. By avoiding the payload inspection we are able to overcome the difficulty of handling encrypted payloads and also avoid compromising the privacy of individual users. We combine these unique bot specific features with the flow statistics of the node to obtain the network profile of a node. Third, differentiating between the behavior of a normal node and a P2P bot is a complex problem. Towards this, we use machine learning techniques to cluster and classify the collected network profile features. We use the decision tree technique because of its efficiency and ease of implementation. To evaluate our approach, we use real-life data sets which contain a mix of malicious and nonmalicious data. We ensure that the nonmalicious data dominates the malicious content in order to estimate the sensitivity of our approach.

### 1.5. Key Contributions

The key contributions of our work are as follows. (a) We describe the first node-based approach to detect P2P bots in a P2P botnet. Our approach is a significant deviation from the signature-based and flow-based detection approaches. (b) We describe a sampling technique to reduce the overhead of monitoring for the network administrator. (c) We abstract and quantify the network profile features of a P2P bot. Our abstraction technique avoids dealing with issues like packet encryption and user privacy. (d) We describe the use of efficient machine learning algorithms to classify the network profile into normal and P2P bot profile. (e) We have evaluated our approach on real-life malicious traffic datasets and obtained a detection accuracy of 99-100% with extremely low false positives in the range of 0–2%. We also show that existing state-of-the-art techniques perform poorly on the same data set when compared to our approach.

#### 1.5.1. Organization

In Section 2, we describe the related research in this domain. In Section 3, we describe our node-based detection approach. In Section 4, we perform a detailed evaluation of our approach. We compare our scheme with other existing approaches in this domain. We summarize our paper and describe future directions in Section 5.

## 2. Related Research

Signature-based bot detection approach has been widely studied [7–13]. This approach is effective to detect known bots, for example, Phatbot. Kolbitsch et al. [19] proposed a signature-based malware detection system which uses special graphs to detect different kinds of bots. However, the detection rate in this approach is only 64%. The utility of signature-based methods is limited as they are not capable of detecting unknown bots or variants of known bots. In the current Internet scenario numerous new bot variants are increasing rapidly, thereby necessitating the need for more adaptive approaches for bot detection.

Flow-based analysis for bot detection has better detection rate. These techniques [14, 15] were proposed to model a wider range of bot behaviors than those covered in signature-based techniques. Livadas et al. [16] developed a system to detect C&C traffic of botnets based on flow analysis. This system consists of two stages: the first stage extracts several per-flow traffic features including flow duration, maximum initial congestion window, and average byte count per packet; and the second stage uses a Bayesian network classifier to train and detect bots. However, the observed false positive rate is still very high, 15.04%, as it fails to capture botnet specific network profiles effectively. Choi et al. [17] proposed a botnet detection mechanism based on the monitoring of DNS traffic during the connection stage of bots. However, a botnet can easily evade this mechanism, if it rarely uses DNS at its initialization and limits or avoids DNS usage at latter stages.

Wang et al. [20] presented a detection approach of P2P botnets by observing the stability of control flows in the botnet initialization time intervals. However, this approach suffers from high performance and storage overhead while achieving similar detection accuracy as earlier approaches. Kang et al. [15] proposed a novel real-time detection model named the multistream fused model, in which they process different types of packets in a graded manner. However, this model cannot achieve desirable detection accuracy when deployed in a large-scale network environment. Liu et al. [18] presented a general P2P botnet detection model based on macroscopic features of the network streams by utilizing cluster techniques. The proposed method is unreliable or ineffective if only a single infected machine is present on the network.

To the best of our knowledge, there has been no research focusing on the application of node-based analysis for P2P bot detection The node-based approach has distinct advantages that separate it from signature-based and flow-based techniques.

## 3. Node-Based P2P Bot Detection

In this section, we describe our node-based P2P bot detection approach. Our approach consists of four important steps: P2P bot quantification, efficient flow monitoring, classification, and evaluation. In Section 3.1, we describe our methodology for modeling P2P bots which is the most important step of our detection approach. Using this model, we identify the features to quantify a P2P bot. In Section 3.2, we describe our approach for reducing the complexity of the flow monitoring at the network nodes. In Section 3.3, we describe our classification approach, for identifying P2P profiles from the set of network profiles of all nodes, and describe the evaluation metrics of the classification approach.

### 3.1. Our Model for Quantifying P2P Bot Features

In our node-based P2P bot detection approach, we monitor the communication flows at every node in the network to check for bot infection. Since each flow can exhibit many features, it is important to identify and isolate features which are unique to P2P bots. Our model of P2P bots is based on two key observations. First, since a P2P bot is part of a P2P network, it exhibits the communication behavior of a normal P2P node but with some distinguishable differences. Second, a P2P bot exhibits different types of network activity compared to regular P2P nodes. Now, using these observations, we identify several important features of a P2P bot. We group the features into two categories, P2P bot communication model and P2P bot behavior model, respectively, and describe them as follows.

#### 3.1.1. P2P Bot Communication Model

In a P2P network, a node might attempt to connect to one or more network peers periodically in order to maintain the connection status or to query for data of interest. A P2P bot performs a similar activity but with the key difference being that the P2P bot attempts such connections more actively so as to ensure the connectivity across the P2P botnet. This behavior is uniform across all P2P bots in the P2P botnet. Furthermore, unlike regular P2P communication, where the P2P node attempts connections based on responses received from other peers, the P2P bot attempts to initiate connections proactively. Therefore, at the beginning of its activity, a bot sends connection requests to other bot nodes according to the peer list. A certain amount of such requests fails, because some peers are shutdown or not infected. On the contrary, the success rate is usually high when normal P2P applications send connection requests. Thus, the success rate of connection requests is an important criterion for P2P bot detection. From the observed P2P botnet data, we note that if the success rate of a node connection attempt is below 50%, the node tends to be a bot.

#### 3.1.2. P2P Bot Behavior Model

To understand the unique features of P2P botnets, we chose four kinds of real P2P bots available in the wild. Using a controlled virtual environment, with the help of VMware technology, we analyzed these bots. A summary of the results is shown in Table 1. Using this data, we identify the following network features of interest specific to P2P bot behavior. A feature represents a characteristic of a node in a given time window T, which is chosen by the system analyst performing the P2P bot detection.

*Number of Different Protocols.* A majority of the P2P bots utilize both UDP and TCP packets in the same flow, that is, send and receive to the same destination port and address from the same source port and source address. For instance, Bot 2 makes SMTP connection using TCP and sends several UDP packets in the same flow. The more important aspect is that the UDP packets outnumber the TCP packets, which is indicative of P2P bot behavior. 

*Large Number of Flows*. The number of flows in a P2P bot is higher than for a normal P2P node. For instance, in Bot 3, there is a high amount of ICMP traffic from the P2P bot towards port 53 of random IP addresses on the Internet. These packets correspond to discovery packets intended to locate new targets for the P2P bot infection. The number of flows reflects the degree of extensive connections with other nodes. 

*Large Number of Packets*. In P2P botnets, due to the P2P topology, there are a large number of packets exchanged among the P2P nodes. This differs from a server-client botnet where the communication among client nodes is minimal or nonexistent. Therefore, a P2P botnet typically generates a higher average in the number of packets exchanged. This behavior is observed uniformly across all the bots we have analyzed. 

*Average Packet Length*. Since the P2P bots need to exchange updates or instructions regularly with other bots, the size of the packets is necessarily small to avoid detection by the IDS system. However, this results in many continuous uniform small packets which is a useful feature for P2P bot detection. For instance in Bots 1, 2, and 3, there is constant communication among peers regarding P2P status and other features. 

*Ratios of Packets Exchanged*. The number of packets sent and received by P2P bots exhibits certain uniformity across all the bots. These values differ considerably from the regular P2P communication as observed from our analysis of the sample bots. One important feature of interest is ratio of number of packets sent to the number of packets received, RNP, where a higher value of RNP indicates that these nodes are more active than other nodes. Another feature of importance is ratio of the average of length of packets sent to the average of length of packets received, RLP, where the value of RLP is an indicator to the peering relationship between nodes, a lower value indicating a normal P2P node and a higher value indicating a P2P node controlled by some other peer nodes.

Table 2 lists the seven features we have selected for the purposes of P2P bot detection. In this list, the features, such as the source and destination IP addresses, are extracted directly from the TCP/UDP headers. Other features, such as the number of protocols used, require additional processing and computation. Therefore, we perform flow monitoring on individual nodes to extract the desired features. We describe our flow monitoring approach next.

### 3.2. Flow Monitoring

Our node-based detection approach monitors the flows at each node to extract the network features identified in Table 2. We note that, even with this requirement, the node-based analysis is still much more efficient than signature-based analysis. But there are two challenges in flow monitoring. First, analyzing each flow at a node requires capturing each packet. The process of packet capturing is known to suffer from high packet losses. Specialized hardware might be required to handle the packet loss which may prove to be an expensive option. Second, as some features, like NFS, NP, RNP, RLP and so forth, cannot be obtained from packet headers directly, the packets need to be stored and processed. This results in a large storage overhead for the bot detection process. To overcome these two challenges, we adopt the sampling approach proposed in [21, 22].

**Figure 4:**
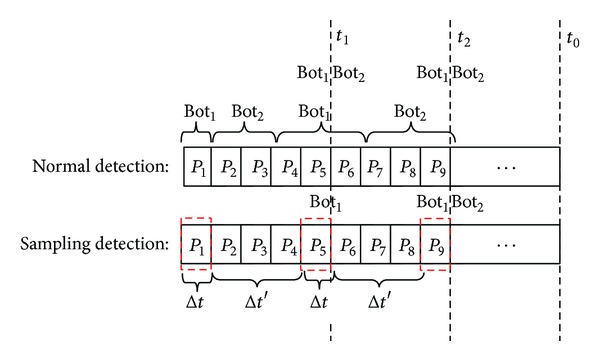
Effect of sampling on bot detection.

Specifically, for each flow at the node, we sample the packets in a periodic manner, thereby reducing the number of packets that need to be captured. However, reducing the number of captured packets can reduce accuracy of bot detection. To evaluate the impact of sampling on bot detection, we compare continuous packet capturing against sampling and show the results in Figure 4. This figure shows that the normal capturing can possibly detect more bots than sampling detection within the same time window. For example, at around time t1, the normal packet capturing detects two bots while the sampling detects one bot. However, eventually the two methods detect the same number of bots after a few time windows. For example, at around time t2, both normal capturing and the sampling detect two bots. The asymptotic results are possible due to the cycle limit, that is, the constant reassignment of the C&C server to different P2P bots.

Therefore, using sampling in combination with our bot detection approach can reduce the overhead of flow monitoring without sacrificing the detection accuracy when considered over a time period. We note that the trade-off in terms of detection time is reasonable as our approach detects all P2P bots within acceptable time windows.

### 3.3. Classification Technique and Evaluation Metrics

For our P2P bot detection approach, we require classification techniques which have high performance in order to support real-time detection goals and at the same time have high detection accuracy. Machine learning classification techniques attempt to cluster and classify data based on feature sets. We have selected the decision tree classifier technique for our evaluation. Decision tree based classifiers exhibit desirably low computational complexity with high performance. In a decision tree, interior nodes represent input features with edges extending from them which correspond to possible values of the features. These edges eventually lead to a leaf node which represents an output variable corresponding to a decision. For our approach, the decision tree is trained based on the real-life P2P bot data using the feature set from Table 2. During the detection phase, the feature set extracted from node's flow information is given to the classifier which essentially classifies this feature set into malicious or nonmalicious feature set.

We consider the standard metrics true positive, TP, true negative, TN, false positive, FP, and false negative, FN, with respect to the classification of feature set into malicious or nonmalicious. The TP and TN values indicate the number of feature sets correctly classified as malicious and benign, respectively. The values FP and FN indicate the number of feature vectors incorrectly classified as malicious and benign, respectively. The true positive rate, TPR, and the false positive rate, FPR, are calculated using the following equations. We define the detection accuracy of our technique using the term, detection rate, DR, and set it to TPR. The true positive rate, TPR, estimates the performance of our P2P botnet detection technique in terms of the probability of a suspicious feature set correctly classified as malicious. On the contrary, the false positive rate, FPR, estimates the probability of a normal traffic being classified as malicious. Finally, we use the standard variable precision to indicate the probability of detection precision of our technique:

(1) DR=TPR=TPTP+FNFPR=FPFP+TNPrecision=TPTP+FP.

We note that the detection rate, DR, approaches 1 if the false negatives, FN, tend to zero. Similarly, the precision approaches 1 if the false positives, FP, tend to zero. Therefore, both the detection rate and the precision have equivalent importance in the P2P bot detection process.

## 4. Experimental Evaluation

In this section, we describe the evaluation results of our node-based P2P bot detection approach. First, we present the performance results of our scheme showing the detection rate and the precision achieved. Next, we compare our approach with general flow-based detection approach and a state-of-the-art detection tool Bothunter [23] which uses event correlation based analysis.

### 4.1. Experimental Methodology and Implementation

We construct our experimental dataset by combining two separate datasets, one containing malicious traffic related to the Storm botnet and the other containing malicious traffic related to the Waledac botnet, obtained from the French chapter of the honeynet project [24]. Waledac is currently one of the most prevalent P2P botnets and has a highly decentralized communication structure than the Storm botnet. While Storm uses Overnet for P2P communication, Waledac utilizes HTTP communication and a fast-flux based DNS network extensively. The highly distributed nature of Waledac makes it resilient to bot detection approaches. Next, we incorporated two benign datasets into our experimental dataset; one is from the Traffic Lab at Ericsson Research in Hungary [25] and the other is from the Lawrence Berkeley National Lab (LBNL) [26]. The Ericsson Lab dataset contains a large amount of traffic from different applications, including HTTP web browsing behaviors, World of Warcraft gaming packets, and from popular bit-torrent clients such as Azureus. The LBNL trace data provides additional nonmalicious background traffic. As the LBNL is a research institute with a medium-sized enterprise network, their trace data provides a different variety of benign traffic such as web, email, network backup, and streaming media data. This variety of traffic serves as a good example of modeling the day-to-day use of enterprise networks.

We implemented our approach in Java. Our program extracts all node information from a given packet capture (pcap) file, and parses the individual node information into relevant features for use in classification. For classification, we utilized the popular Weka machine learning framework [21] with the decision tree instantiation on our data. We used the standard training approach for training and testing our solution. The key intuition is that the feature vectors correspond to individual node flows and the analysis is done based on these features.

### 4.2. Performance of Our Approach

To evaluate our approach, we used different time windows to represent the amount of flow data analyzed; that is, in a wider time window we analyze more flow data. The time window attempts to align to the bot life cycle, that is, to capture the entire bot specific network activity. While it might seem that larger time windows are preferred, our results show that with reasonable time windows we are able to achieve 99-100% detection rates. We have tested our approach using regular and sampled monitoring approaches for flow capture. We have tabulated the values of detection rate, DR, false positive rate, FPR, and precision in Table 3. We selected time windows of 10, 20, 30, 60, and 180 seconds. For regular flow analysis, the time interval of capture is set to 0 seconds; that is, all packets are captured and analyzed. For sampled flow capturing, we set the time intervals of capturing to 10, 20, 60, and 180 seconds. With large time windows, our sampled approach reduces the processing overhead considerably while retaining the detection accuracy near to optimal.

From Table 3, we make an important observation that for both regular and sampled monitoring the average bot detection rate of our approach is 99%. For regular monitoring, which is the row corresponding to time interval of 0 seconds, there is a gradual reduction in the false positive rate as the time window increases and for the time window of 180 seconds, the FPR falls to 0 and the DR and precision values achieved are 1. This result shows that our approach is capable of detecting P2P bots with 100% accuracy given a sufficient time window. This result combined with the 99% accuracy achieved in other time windows validates our node-based bot detection approach.

**Figure 5:**
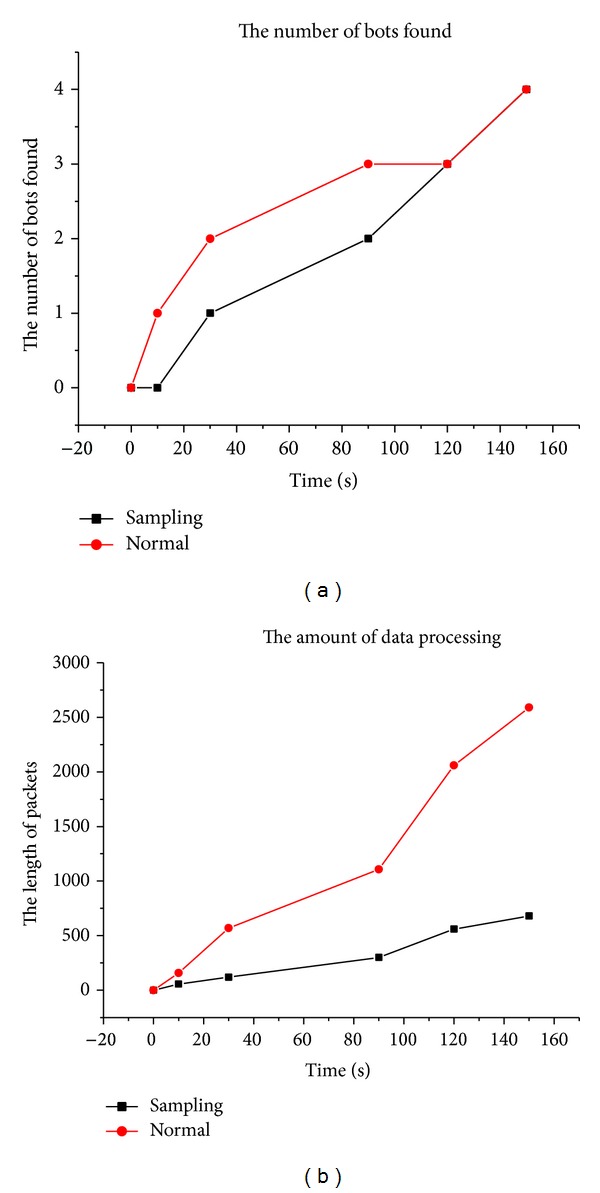
Bots detected in different time windows and data processed for detection.

For sampled monitoring we chose the time intervals of 0, 10, 20, 30, 60, and 180 seconds. From the results in Table 3, we observe that the sampling has limited effect on DR and precision while the FPR shows a slight increase for smaller time windows. However, the impact of FP on precision is very slight. These results show that our bot detection approach works equally well with sampled flow monitoring and hence can scale to real-time detection for high performance systems. The main advantage of sampling is that it reduces more than 60% of the input raw packet traces while retaining the high detection rates and very low false positive rates 0–2% when viewed in the absolute terms. The number of bots found in different time windows and the length of packets captured are illustrated in Figure 5. We can see from this figure that the amount of data processed, denoted by the length of packets, is considerably smaller for sampling detection while detecting the same number of bots.

### 4.3. Comparison with Flow-Based Detection

We compared our node-based approach with the generic flow-based bot detection approaches [15–18, 20, 27]. Since our node-based approach has broader adaptability to new bot behaviors, we expected our approach to perform better than flow-based approaches. To verify this expectation, we implemented flow-based detection by extracting 12 features from the network flows as shown in Table 4. The summary of our experimental results is shown in Table 5. Our approach has lower false positive rate than flow-based approach and the detection rate is higher. More importantly, our approach has better performance since the sampled detection approach reduces the processing and storage overhead considerably.

### 4.4. Comparison with BotHunter

BotHunter [23] is one of the few botnet detection tools relevant to our work and is publicly accessible. BotHunter mainly consists of a correlation engine that creates associations among alerts generated by Snort [22]. For generating Snort alerts, Bothunter uses two custom plugins, the SLADE plugin for detecting payload anomalies and the SCADE plugin for detecting in/out bound scanning of the network. In addition to regular Snort rule sets, Bothunter uses an enhanced rule set that is specifically designed to detect malicious traffic related to botnet activities, such as egg downloads and C&C traffic. The correlation engine analyzes all the alerts, creates associations among them, and generates a report for botnet infections.

When we tested BotHunter on our dataset, the generated alerts indicated that there is a spambot in the dataset. More specifically, there were three alerts with priority 1 that reported the presence of botnet traffic. But all three alerts pointed to the same IP address corresponding to a machine infected with the Waledac botnet. Moreover BotHunter failed to detect the other machine that was infected with the Storm botnet. Finally, among the 97,043 unique malicious flows in the system, BotHunter was able to detect only 56 flows which is a very small percentage of the total flows. These results show that our approach performs better than BotHunter in terms of both performance and detection accuracy.

## 5. Conclusion and Future Work

We described node-based detection, a novel direction to detect P2P botnets. Our approach is node-centric and focuses on modeling the network behavior of individual nodes. Our model is constructed by using a combination of the P2P communication model and the observed behavior of real-life P2P botnets. We identified useful features that are indicative of bot behavior and extract these features using the flows at individual nodes. Due to the generality of our P2P bot model, we are able to use sampling to reduce the effort of flow monitoring at individual nodes while retaining high detection accuracy. Since our model is based on observed behavior, our approach is resilient to variations in protocols and payload obfuscations usually employed by P2P botnets. Our experimental results over different time windows show that by choosing an appropriate time window we can achieve 99-100% accuracy of detection. We have also shown that our approach outperforms existing approaches considerably.

We note that it is very important and necessary to design a system that can evaluate the performance of the detection online instead of the present off-line mechanism in our work. It is also important to train the detection system online, instead of an off-line training process so that it is suitable for live deployment. Such a system is ideal for identifying new threats such as zero-day malware. We will explore these issues in our future work. Further, we will explore the use of AIS, Artificial Immune System, to solve the huge number of behavior problems and identify key influence factors in the future.

## Figures and Tables

**Table 1:** Sample bot analysis and behavior.

Name	Host behavior	Network behavior	Remark
Bot 1. Phatbot	(1) Modify the registry (2) Add startup item (3) Modify a file (4) Terminate antivirus thread	(1) Start the IRC thread (2) Start the P2P server thread (3) Start the P2P client thread	(1) Modify a file named host in system directory (2) Start the thread of IRC client, and connect to IRC server. (3) In order to improve the communication of p2p, start both client thread and server thread

Bot 2. Zhelatin .zy	(1) Modify the registry (2) Add a startup item (3) Copy file	(1) Connect to SMTP server (2) UDP connection	(1) In order to a bot's propagation, copy the bot itself to the shared directory (2) Connect to SMTP Server by SMTP thread (3) A lot of UDP connections with both the same source port and the random target port

Bot 3. Sinit		(1) UDP protocol (2) A high ICMP traffic (3) Sending packets to port 53	(1) Sending special discovery packets to port 53 of random IP addresses on the Internet.

Bot 4. Nugache	(1) Modify the registry	(1) Open TCP port 8 (2) Encrypted data transmission	(1) Modify the registry and install the list with hosts into Windows's registry. (2) Has a static list of IP addresses (20 initial peers) to which it will try to connect on TCP port 8. (3) The exchanged data is encrypted.

**Table 2:** Features for node-based analysis.

Feature	Description
(1) Node	Computer address for transmitting information
(2) NP	Number of protocols used for time interval
(3) NF	Number of flows used for time interval
(4) NPS	Number of packets sent for time interval
(5) ALPS	Average length of packets sent
(6) RNP	Ratio of number of packets sent to number of packets received for time interval
(7) RLP	Ratio of average sending packets length to average receiving packets length for time interval

**Table 3:** Detection rate and precision of node-based detection.

Time interval	Time window	Detection rate	FPR	Precision
0	10	0.997	0.026	0.997
	20	0.998	0.024	0.998
	30	0.998	0.007	0.997
	60	0.999	0	0.999
	180	1	0	1

10	10	0.998	0.004	0.998
	20	0.998	0	0.997
	30	0.999	0	0.998
	60	0.995	0	0.995
	180	0.996	0	0.996

20	10	0.998	0.005	0.998
	20	0.997	0.035	0.996
	30	0.999	0.015	0.998
	60	0.997	0	0.996
	180	1	0	1

30	10	0.998	0.016	0.998
	20	0.998	0.007	0.998
	30	0.997	0.053	0.997
	60	0.999	0	0.998
	180	1	0	1

60	10	0.998	0.053	0.997
	20	0.999	0	0.998
	30	0.997	0	0.997
	60	0.999	0.01	0.998
	180	0.999	0	0.998

180	10	1	0	1
	20	1	0	1
	30	1	0	1
	60	0.999	0.026	0.999
	180	1	0	1

**Table 4:** Features for flow-based detection comparison.

Attribute	Description
SrcIp	Flow source IP address
SrcPort	Flow source port address
DstIp	Flow destination IP address
DstPort	Flow destination port address
Protocol	Transport layer protocol or “mixed”
APL	Average payload packet length for time interval
PV	Variance of payload packet length for time interval
PX	Number of packets exchanged for time interval
PPS	Number of packets exchanged per second in time interval *T*
FPS	The size of the first packet in the flow
TBP	The average time between packets in time interval
NR	The number of reconnections for a flow
FPH	Number of flows from this address over the total number of flows generated per hour

**Table 5:** Comparison of flow-based and our approach.

Attribute	Flow-based	Our approach
True positive rate	98.3%	100%
False positive rate	0.01%	0%
